# UV/Vis Spectroelectrochemistry as a Tool for Monitoring the Fabrication of Sensors Based on Silver Nanoparticle Modified Electrodes

**DOI:** 10.3390/s130505700

**Published:** 2013-05-02

**Authors:** Cristina Fernández-Blanco, Álvaro Colina, Aránzazu Heras

**Affiliations:** Department of Chemistry, Universidad de Burgos, Pza. Misael Bañuelos s/n, E-09001 Burgos, Spain; E-Mails: acfernandez@ubu.es (C.F.-B.); acolina@ubu.es (Á.C.)

**Keywords:** UV-Vis spectroelectrochemistry, silver nanoparticles, screen-printed electrodes, electrochemistry

## Abstract

A new controlled current multipulse methodology has been developed to modify the screen-printed electrode surface with silver nanoparticles (AgNPs). Spectroelectrochemistry has provided not only information about the type of nanoparticles (NPs) deposited on the electrode surface, but also about the electrosynthesis process. Small NPs without plasmon band are initially generated. Next, these nuclei grow to form bigger NPs in the reduction pulses with a characteristic plasmon band centered at 400 nm. Most of the NPs are generated during the first reduction pulses and a linear growth of the absorbance at a lower reaction rate was obtained in the subsequent pulses. Oxidation pulses do not redissolve completely silver NPs but only partially, meaning that very stable NPs are generated. AgNPs-modified electrodes have been successfully used to determine hydrogen peroxide. Spectroelectrochemistry has also yielded very useful information to understand the voltammetric signal obtained during the reduction of H_2_O_2_ on silver modified electrodes.

## 1. Introduction

Metal nanoparticle-modified electrodes have been widely used as electrochemical sensors for determination of very different compounds [1–3]. In principle, electrodeposition of metal NPs is a very simple way to modify electrodes [4–8], however, sometimes developing a new electrochemical synthetic route to generate NPs in a reproducible way is not as easy as might be expected. Moreover, we need to use high resolution microscopy techniques to be sure that our electrode has been successfully modified. These microscopy techniques are expensive and not readily available for many researchers. A good alternative to obtain information on NPs modified electrodes is UV/Vis spectroelectrochemistry. Our group has recently published a work about the electrodeposition of gold NPs on Pt electrodes [9]. UV/Vis spectroelectrochemistry has been demonstrated to provide suitable information on the NPs generated on the electrode surface, that is comparable to that obtained by scanning electron microscopy [9]. Spectroelectrochemistry is a multiresponse technique that has been used to study complex reaction mechanisms [10–12]. It yields *in-situ* spectral information during the electrodeposition of the NPs on the electrode, which allows us to know the type of NPs deposited on the electrode, providing a fast and low-cost analytical method to assure that the electrode has been successfully modified with NPs. This hybrid technique is extremely useful in the electrosynthesis of NPs like gold or silver that have a characteristic plasmon band, whose position, width and shape is directly related to the kind of NPs obtained. For example, spectra recorded during gold electrodeposition were compared with SEM images of the deposited NPs and a good correlation was obtained between both techniques [9]. AgNPs exhibit a plasmon band that can be easily followed using spectroscopy during the electrodeposition [13–15]. Position and intensity of the plasmon band depends on the shape, size and density of the AgNPs. Therefore, spectroelectrochemistry is a very useful technique to study this electrodeposition process.

During the last years a number of authors have shown that AgNPs exhibited remarkable electrocatalytic activity for the reduction of hydrogen peroxide [16–22]. The marked acceleration of this redox process is very attractive for developing new electrochemical sensors. Hydrogen peroxide is a very important compound that is used in very different applications such as chemical synthesis, environmental processes, cleaning products, and the paper, food and textile industries [19,20,23].

Screen printed electrodes (SPEs) have been used as disposable electrochemical sensors [24–26]. In a recent work we developed a new methodology to perform spectroelectrochemical measurements on SPEs [27]. The same methodology has been used in this work to follow the electrochemical synthesis of AgNPs on SPE. The main objective of this work was to develop a new AgNPs synthesis route based on galvanostatic multipulse techniques, and demonstrating that spectroelectrochemistry can be used to follow *in-situ* the electrodeposition process. The AgNPs-modified electrode has then been used as a sensor for amperometric detection of hydrogen peroxide. Besides, spectroelectrochemistry has been also used to understand the electrochemical signal obtained during the H_2_O_2_ reduction.

## 2. Experimental Section

### 2.1. Chemicals

All solutions were prepared fresh daily from analytical reagent grade chemicals using high-quality water (resistivity of 18.2 MΩ·cm, Milli-Q A10 system, Millipore, Billerica, MA, USA). Silver nitrate (AgNO_3_, Acros Organics, Geel, Belgium), hydrogen peroxide (H_2_O_2_, Merck, Darmstadt, Germany), potassium dihydrogen phosphate (Merck, Darmstadt, Germany) and sodium hydrogen phosphate (Merck, Darmstadt, Germany) were used as received. 4 × 10^−3^ M AgNO_3_ aqueous solutions were prepared to synthesize AgNPs. The supporting electrolyte for H_2_O_2_ solutions was a phosphate buffer solution (PBS), pH = 7, containing 0.2 M Na_2_HPO_4_-KH_2_PO_4_.

### 2.2. Instrumentation

Spectroelectrochemical experiments were carried out at room temperature using the previously described experimental set-up [27]. All experiments were performed using commercial SPEs (DRP-550, DropSens S.L., Llanera, Asturias, Spain) with the three electrodes printed on the same strip. The strips had a 4 mm diameter disk screen-printed platinum working electrode (WE), a platinum counter electrode (CE) and a silver pseudo-reference electrode (RE). SPEs were placed in a box connector DSC (DropSens S.L., Llanera, Asturias, Spain) that held the SPE and allowed us to reproduce its position between measurements.

An AUTOLAB PGSTAT 10 potentiostat/galvanostat electrochemical system (Metrohm Autolab B.V., Utrecht, The Netherlands) coupled with a QE65000 Spectrometer (Ocean Optics Inc, Dunedin, FL, USA) that consists of a 2D diode array of 1,044 × 64 pixels were used to perform the spectroelectrochemical experiments. A PEEK reflection UV/Vis probe (FCR-7UV200, Avantes B.V., Apeldoorn, The Netherlands) was used to conduct the light beam from the deuterium-halogen light source (Avalight-DH-S, Avantes B.V., Apeldoorn, The Netherlands) to the spectroelectrochemical cell, and from the spectroelectrochemical cell to the spectrometer. The reflection probe was placed in a home-made cell [27] facing the surface of the screen-printed Pt working electrode at a distance of approximately 1.25 mm.

## 3. Results and Discussion

### 3.1. Modification of SPEs with AgNPs: Spectroelectrochemical Analysis

**Figure 1.**
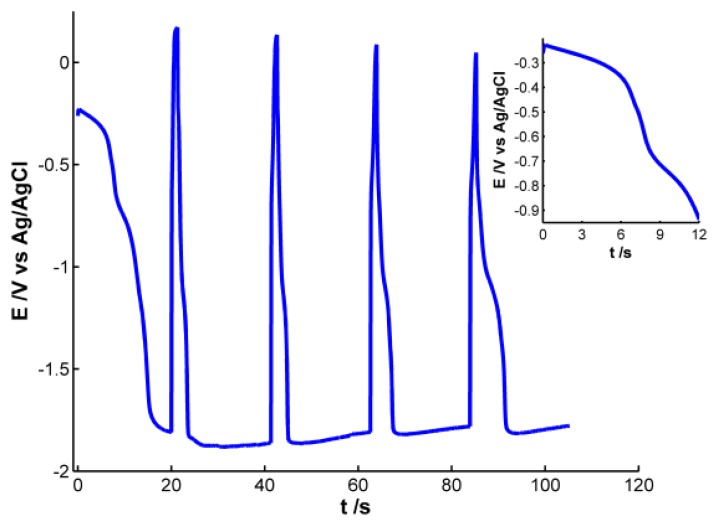
Chronopotentiogram obtained during the galvanostatic multipulse synthesis of AgNPs applying nine reduction/oxidation currents of −1 × 10^−4^ A for 20 s and 1 × 10^−4^ A for 1.3 s. (Inset: Zoom corresponding to the first 12 s of the galvanostatic experiment).

Pt working electrode of the SPE was modified with electrochemically synthesized AgNPs. Different experiments were carried out to obtain AgNPs showing a well-defined plasmon band. For this purpose, we used a galvanostatic multipulse strategy that allowed us to have a better control of the deposition of NPs on the electrode surface. After the optimization of the experimental variables the following electrochemical conditions were selected: a chronopotentiometric experiment that consists of nine steps alternating a reduction current of −1 × 10^−4^ A for 20 s with an oxidation current of 1 × 10^−4^ A for 1.3 s was performed in a 4 × 10^−3^ M AgNO_3_ aqueous solution. Figure 1 shows the chronopotentiometric response obtained during electrosynthesis of AgNPs.

During reduction pulses a decrease of potential is observed, indicating that silver is being reduced and deposited on the electrode surface. An almost stable potential value around −1.80 V is achieved in the four last reduction steps, indicating that this is the lowest potential value at which silver reduction occurs under these experimental conditions. On the other hand, during oxidizing steps a drastic increase of potential is observed, revealing that some of the silver atoms are oxidized instantaneously. Even though potential changes indicate that silver ions are being reduced during reduction steps and silver is being oxidized to some extent during oxidation pulses, only poor information can be extracted from the electrical response. It is not possible to obtain any conclusive information about the generation of AgNPs on the electrode surface, so simultaneously to the chronopotentiometric experiments, UV-Visible spectra were recorded *in-situ*.

**Figure 2.**
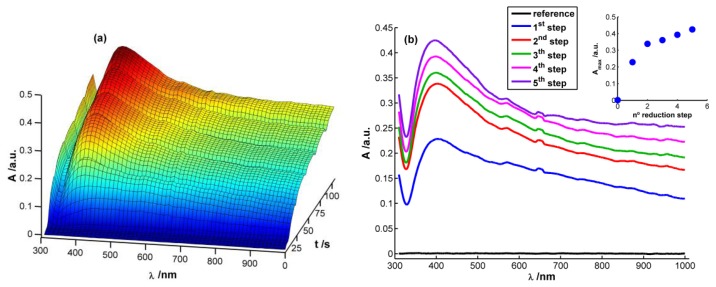
(**a**) UV/Vis spectra temporal evolution during the galvanostatic multipulse synthesis of Ag NPs. (**b**) Spectra at the end of each reduction step (inset: maximum of absorbance at the end of each reduction step). Experimental conditions as in Figure 1.

Figure 2(a) displays a 3D image corresponding to the evolution of absorbance spectra with time during the chronopotentiometric experiment. In this case, each spectrum between 310 and 1,000 nm was recorded each 0.1 s, taking as reference spectrum the one obtained just before starting the multipulse experiment. As can be observed, a characteristic plasmon band grows centered at 400 nm that, undoubtedly, can be related to the presence of AgNPs on the WE surface [13,15,28]. This band grows during each reduction pulse (Figure 2(b)). Taking the absorbance at 400 nm at the end of each reduction step (A_400_) as a way to evaluate AgNPs growth process, it can be observed that the higher increment of A_400_ takes place during the first and second reduction steps. During the next ones a linear increase of absorbance is observed (R^2^ = 0.9931), meaning that a constant reaction rate is achieved from the second reduction step onwards (inset in Figure 2(b)). It is noteworthy that the reaction rate is approximately eight times lower from the second reduction step onwards than during the first reduction pulse, implying a much higher reaction yield in the first reduction pulse than in the following ones.

Performing a more exhaustive analysis of the spectra evolution, a global increment of absorbance can be observed during the first seconds of the experiment after applying the reduction current in the whole spectral range between 310 and 1,000 nm (Figure 2(a)). This unshaped spectrum is characteristic of small AgNPs (<2 nm) [28], which can be related to the first nuclei generated on the electrode surface. Next, a well-defined plasmon band starts to emerge around 400 nm (Figure 2(a)). Absorbance is significantly different from zero at the higher wavelengths of the spectra once the plasmon band emerges indicating that monodispersity is not achieved. Spectra suggest that on the WE surface are deposited both AgNPs with plasmon band and smaller AgNPs without a characteristic plasmon band.

**Figure 3.**
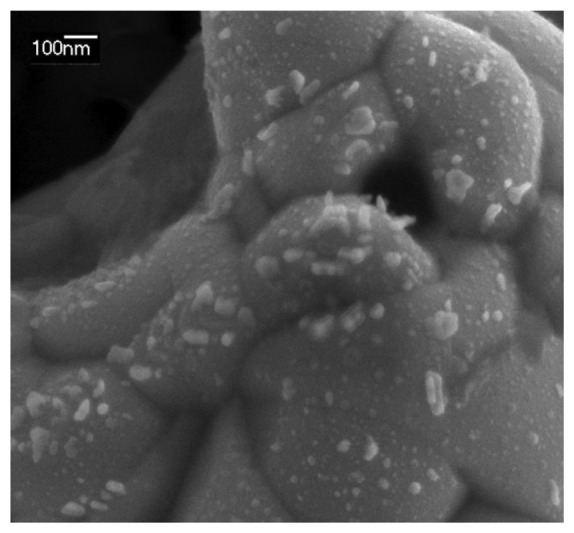
SEM image of AgNPs synthesized by the galvanostatic multipulse method described.

A SEM image of the AgNPs galvanostatically synthesized on the SPE is shown in Figure 3. Although some big AgNPs of around 100 nm are observed, most of the Pt surface is covered with 10–20 nm AgNPs. This image confirms that AgNPs have been deposited on the electrode surface, as was observed by spectroelectrochemistry in a much faster and cheaper way.

**Figure 4.**
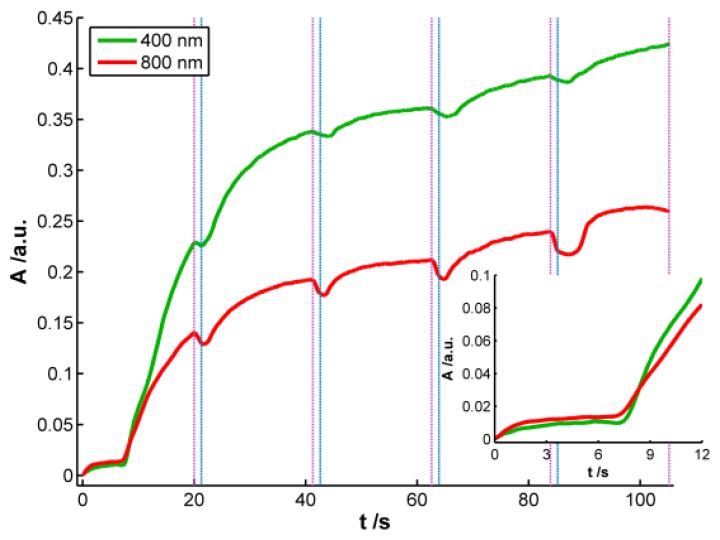
Absorbance evolution during the galvanostatic multipulse synthesis of AgNPs at ( 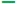) 400 and ( 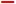) 800 nm. Pink lines ( 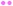) mark the end of each reduction pulse and blue lines ( 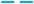) the end of each oxidation pulse. (Inset: Zoom corresponding to the first 12 s of the galvanostatic experiment). Experimental conditions as in Figure 1.

According to the previous discussion, it is possible to establish that two regions should be considered in the spectra registered during AgNPs synthesis. One of these spectral regions is related to 10–20 nm AgNPs. The other one is related to smaller AgNPs that absorb radiation in the whole spectral range and could only be observed by using very high-resolution microscopes. Consistent with the plasmon band waveform, at wavelengths longer than 700 nm the absorbance is not directly related to 10–20 nm AgNPs but rather to smaller NPs (<2 nm) [28]. For this reason 800 nm was chosen as the representative wavelength for this second region. Figure 4 displays the temporal evolution of these two wavelengths, 400 and 800 nm, along the galvanostatic multipulse experiment. From absorbance evolution shown in Figure 4 two different behaviors can be deduced. Initially (inset of Figure 4), a small absorbance increase is observed for both wavelengths during the first two seconds of the experiment, being slightly higher for 800 nm $\left({\text{A}}_{800\;\text{nm}}^{2\;\text{s}}/{\text{A}}_{400\;\text{nm}}^{2\;\text{s}}=1.5\right)$. During the next 5 s absorbance remains almost constant for both wavelengths. Next a sharp increase of absorbance is observed for both wavelengths, with a crossing point appearing at 8.5 s (inset of Figure 4). From this time onwards, absorbance at 400 nm is significantly higher than the one at 800 nm. Just at this time, 8.5 s, the plasmon band corresponding to bigger AgNPs evolves in the UV/Vis spectra. This point corresponds to the first abrupt decrease of potential observed in the chronopotentiogram (inset in Figure 1). From the analysis of the optical and electrical information, we can conclude that during the first 7–8 s of the galvanostatic experiment only smaller AgNPs are electrogenerated. According to bibliography these NPs are smaller than 2 nm [28] and do not exhibit plasmon band. From 8.5 s onwards, bigger AgNPs are electrosynthesized on the electrode surface, that is to say, NPs showing a plasmon band centered at 400 nm. The lower increase of absorbance at 800 nm respect to that at 400 nm indicates that some of the smallest AgNPs are consumed to generate bigger ones. The two processes described correspond to the well-known nucleation and growth steps in nanoparticle synthesis [29].

After each reduction step, in which absorbance increases, an oxidation current that lasts 1.3 s is applied. During these oxidation steps a small decrease of absorbance is observed related to the partial reoxidation of less stable Ag atoms, and as can be seen in Figure 4, AgNPs are not completely redissolved. It should be remarked that the decrease of absorbance is always higher at 800 nm than at 400 nm, indicating that oxidation of small NPs without plasmon band occurs to a higher extent than oxidation of bigger ones with plasmon band centered at 400 nm. As it has been demonstrated before [9], oxidation steps help to obtain a more homogeneous NP size.

As it was stated above and concluded from inset of Figure 2(b), reaction rate is faster in the first reduction pulse than in the following ones. This behavior corresponds with a higher increment of absorbance, both at 400 and 800 nm, in the first reduction pulse than in the next ones. It is also remarkable that absorbance increase during the last 12 s of the first reduction pulse. However, in the last three reduction pulses, absorbance only increases slightly during the first 4–5 s of each pulse. From these absorbance changes it can be concluded that AgNPs are generated mainly in the first reduction pulse, while in the last three ones the small increment of absorbance detected should correspond basically with the reduction of Ag atoms oxidized during the previous anodic pulse. In this three last reduction pulses an almost constant value of absorbance is achieved approximately in the last 15 s, indicating that stable AgNPs strongly bound to the WE surface are generated.

### 3.2. Electrochemical Detection of H_2_O_2_ with AgNPs-Modified SPEs

Once stable AgNPs are formed on the WE surface, the modified electrode has been used as an electrochemical sensor for hydrogen peroxide. Reduction of H_2_O_2_ takes place at lower overpotential on the AgNPs-modified electrode than on a pristine Pt electrode, demonstrating the catalytic effect of these NPs. Seven H_2_O_2_ samples were prepared in the range from 0 to 11 × 10^−3^ M in buffer solution (pH = 7). We have also performed spectroelectrochemical experiments using the modified SPEs to shed more light on the reduction process of H_2_O_2_. Cyclic voltammograms between −0.20 V and −0.75 V at 0.05 V·s^−1^ were carried out to study the reduction process and quantify the hydrogen peroxide. Spectra between 310 and 1,000 nm were registered each 0.1 s, taking the initial spectrum at −0.20 V as reference. The same SPE modified with AgNPs has been used to detect H_2_O_2_ in all samples, cleaning it thoroughly with deionized water between each analysis. H_2_O_2_ does not absorb light in the spectral range studied in these experiments, but changes in the surface of AgNPs deposited on the SPE during H_2_O_2_ reduction can be detected using UV-Visible spectroelectrochemistry.

**Figure 5.**
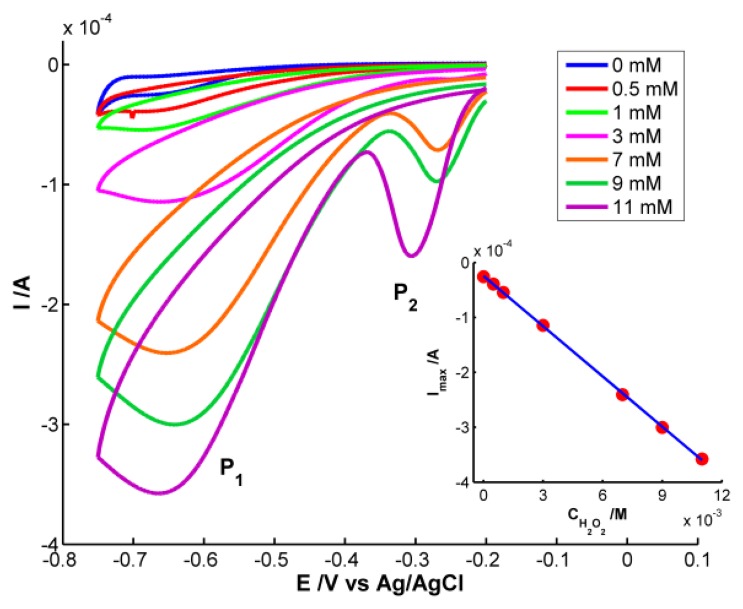
Voltammetric response of AgNPs-modified SPE when H_2_O_2_ (0–11 × 10^−3^ M) was added to buffer solution (pH = 7). Scan rate: 0.05 V·s^−1^; E_initial_ = E_final_ = −0.20 V; E_vertex_ = −0.75 V (inset: calibration curve where current intensity at P_1_ is plotted *versus* H_2_O_2_ concentration)

As could be expected, only the electrical signal is useful for quantitative analysis because H_2_O_2_ does not absorb in the spectral range. Cyclic voltammograms are shown in Figure 5. Significant differences in the voltammogram waveform are observed for those obtained at H_2_O_2_ concentrations higher and lower than 3 × 10^−3^ M. At low hydrogen peroxide concentrations, only one reduction peak (P_1_) is observed at potentials around −0.68 V. This peak is related to the reduction of H_2_O_2_ to H_2_O and O_2_[20]. However two reduction peaks were observed at H_2_O_2_ concentrations higher than 3 × 10^−3^ M. One peak (P_1_) is around −0.68 V and a new one (P_2_) between −0.27 and −0.31 V. P_2_ has been linked to an “activated” mechanism in which OH ions are adsorbed on the AgNPs surface, as some authors have explained [21,22].

**Figure 6.**
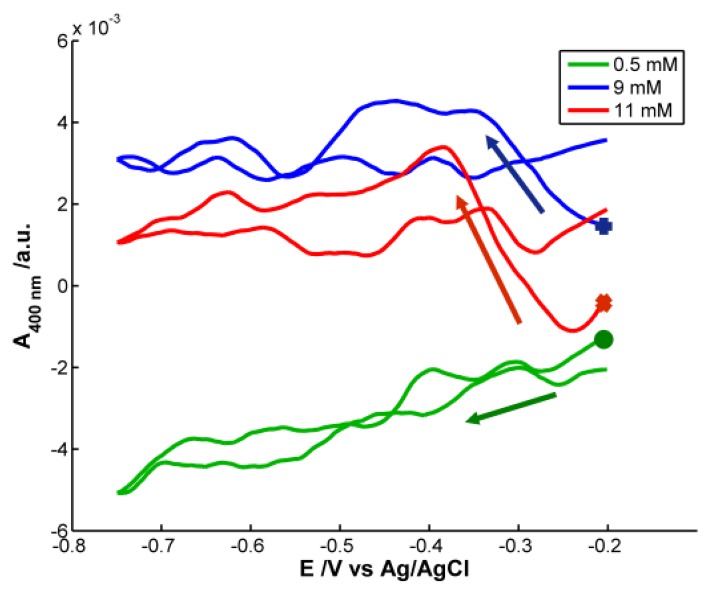
Voltabsorptogram at 400 nm registered during H_2_O_2_ reduction on AgNPs-modified SPE. ( 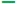) C_H2O2_ = 5 × 10^−4^ M, ( 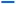) C_H2O2_ = 9 × 10^−3^ M, ( 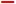) C_H2O2_ = 1.1 × 10^−2^ M. The following symbols ( 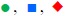) mark the first absorbance value in each voltabsorptogram. Arrows mark the direction of the reduction scan. Experimental conditions as in Figure 5.

Although H_2_O_2_ does not absorb in the spectral range, the optical signal provides very interesting information. Analyzing the spectra obtained during the voltammetric experiments, some differences are observed depending on the concentration of H_2_O_2_. Figure 6 represents the voltabsorptograms at 400 nm, the characteristic wavelength of 20–30 nm AgNPs previously synthesized.

At the lowest H_2_O_2_ concentration, 5 × 10^−4^ M, very small absorbance changes are observed. During the reduction scan absorbance at 400 nm decrease 0.004 a.u. recovering its initial value in the backward scan. No significant effect of the potential during the reduction of H_2_O_2_ is observed on the NPs deposited on the SPE. However, in the case of the highest H_2_O_2_ concentration, 1.1 × 10^−2^ M, around −0.25 V, absorbance at 400 nm abruptly increases until a potential of −0.40 V is reached. This potential interval coincides with the reduction peak that evolves in the voltammogram for H_2_O_2_ concentration of 1.1 × 10^−2^ M around −0.30 V (P_2_). A deeper analysis of voltammograms evidences that this reduction peak (P_2_) shifts to less negative potentials when decreasing H_2_O_2_ concentrations. For example, at 9 × 10^−3^ M, P_2_ is around −0.27 V. The same shift is observed in the voltabsorptogram registered for this solution at 400 nm. The absorbance increment takes place between −0.20 V and −0.33 V, a potential range that includes the position at which the cathodic peak P_2_ is observed in the cyclic voltammogram. Taking into account the change of absorbance at 400 nm, detected for the more concentrated solutions of H_2_O_2_, considering that H_2_O_2_ does not absorb in our spectral range, and knowing that Ag NPs bigger than 2 nm have a plasmon band at 400 nm, it can be concluded that the reduction peak P_2_ must be related to the surface reduction of AgNPs deposited on the SPE. The higher the H_2_O_2_ concentration is, the higher AgNPs surface is oxidized by this H_2_O_2_ before starting the voltammetric experiment. Therefore, before starting reduction of H_2_O_2_ at potentials lower than −0.35 V, the surface of AgNPs is re-reduced. The reduction peak P_2_ is not proportional to the H_2_O_2_ concentration, and therefore it is not useful for quantitative analysis.

Finally, quantitative analysis was performed using the calibration set of H_2_O_2_ samples prepared between 0 and 1.1 × 10^−2^ M. Taking the current of the cathodic peak P_1_ as response, the electrochemical calibration curve was constructed: Ip (A) = −3.048 × 10^−2^ × C_H2O2_ (M) − 2.454 × 10^−5^ (inset of Figure 5). The good determination coefficient (R^2^ = 0.9998) and the low residual standard error (S_yx_ = 1.885 × 10^−6^) are indicative of the good correlation between peak current and H_2_O_2_ concentration in the studied range. On the other hand, one sample 5 × 10^−3^ M of H_2_O_2_ also prepared in buffer medium (pH = 7) was used to evaluate the prediction capability of the calibration curve constructed. The concentration estimated lead to a confident interval of (4.90 ± 0.17) × 10^−3^ M with a residual standard deviation (RSD) of 1.4% (n = 7). The capability of detection has been calculated for a probability of false positive (α) and negative (β) of 0.05. A detection limit of 1.98 × 10^−5^ M was obtained for this method using the AgNPs-modified SPE.

From these electrochemical calibration data, we can affirm that the constructed AgNPs-modified SPEs are useful for hydrogen peroxide determination. The performance of the electrodeposited AgNPs is very stable for a long time; the electrode has been used repeatedly without significant changes in its electrochemical behavior.

## 4. Conclusions

A new galvanostatic multipulse strategy has been proposed to electrogenerate AgNPs on Pt SPEs. UV/Vis spectroelectrochemical information registered during NPs electrodeposition allows us to detect two different stages. The first stage is related to electrodeposition of small NPs (<2 nm) without any significant spectral feature, occurring during the first 7–8 s after fixing the first cathodic current to the system. On the other hand, the second reaction stage indicates the growth of AgNPs, evidenced by the plasmon band evolution at 400 nm, implying the consumption of small AgNPs. The generation of these bigger NPs, with the characteristic plasmon band, is higher during the first two reduction pulses than in the next ones. It is just in the last three reduction pulses where a constant reaction rate is achieved.

Spectroelectrochemistry has been demonstrated also to be very useful in the study of H_2_O_2_ reduction on AgNPs-modified SPE. At low H_2_O_2_ concentration only one reduction peak is observed (P_1_), but when H_2_O_2_ concentration is increased, a second reduction peak (P_2_) evolves at potentials less negative than the main one. An increase of absorbance at 400 nm just in the potential range in which this second peak emerges has been observed in the spectroscopic signal. These spectral changes have been related to partial oxidation of AgNPs surface by H_2_O_2_ before electrochemical reduction starts. AgNPs surface is reduced before starting H_2_O_2_ reduction at sufficiently negative potential.

From a quantitative point of view, the modified electrode has been used for electrochemical determination of hydrogen peroxide in a buffer solution (pH = 7). On one hand, the high determination coefficient and low residual standard error of the calibration curve calculated, and, on the other hand, the low residual standard deviation and the narrow confidence interval obtained in the determination of a problem sample lead to the conclusion that the AgNPs-modified SPEs can be used to quantify this analyte. Once we have demonstrated the usefulness of spectroelectrochemistry to obtain information on the electrodeposition process, this technique could be used in future works to synthesize different types of AgNPs in order to obtain the best catalyst for this or other analytes.
